# Survival of conventional dental implants in the edentulous atrophic maxilla in combination with zygomatic implants: a 20-year retrospective study

**DOI:** 10.1186/s40729-022-00425-3

**Published:** 2022-06-15

**Authors:** Luc Vrielinck, Jorden Blok, Constantinus Politis

**Affiliations:** 1 Department of Oral and Maxillofacial Surgery, East-Limburg Hospital, Genk, Belgium; 2 Department of Oral and Maxillofacial Surgery, University Hospitals Leuven, Leuven, Belgium; 3 OMFS IMPATH Research Group, Department of Imaging and Pathology, Faculty of Medicine, Catholic University of Leuven, Leuven, Belgium

**Keywords:** Dental implant, Zygomatic implant, Survival, Atrophic maxilla

## Abstract

**Purpose:**

Implant-supported prosthetic rehabilitation in the resorbed maxilla is a great challenge. The aim of this study was to determine the survival rate of conventional anterior implants placed in combination with zygomatic implants according to the Brånemark technique, and to identify risk factors for implant failure.

**Methods:**

We collected data retrospectively from 72 consecutive patients who received treatment from 1998 to 2018 at our center, according to Brånemark’s original technique. Kaplan–Meier analysis was conducted to assess survival rate, and a survival regression model was used with the patient as the random factor, applying the Weibull distribution.

**Results:**

A total of 236 maxillary anterior implants were included, with a mean follow-up of 12.1 years. Kaplan–Meier analysis showed overall cumulative survival rates of 95.3% at 1 year, 94.8% at 2 years, 93.0% at 5 years, 90.5% at 10 years, 81.6% at 15 years, and 67.7% at 20 years. Survival regression showed an association between bruxism and implant failure as well as implants bearing an overdenture. Implants with length ≤ 10 mm had a significantly lower survival time. No significant association was found between the number of anterior implants and survival rate.

**Conclusions:**

We found acceptable long-term anterior conventional implant survival. Significant risk factors for failure were bruxism, overdentures, and implants shorter than 10 mm.

## Background

Dental implants require sufficient bone volume and density for successful osseointegration. Implant-supported prosthetic rehabilitation in resorbed jawbones thus is a great challenge, especially in the posterior maxilla [1]. Several techniques to manage the resorbed upper jaw have been proposed to improve the stability of dental implants [2–4]. Among these, zygoma-anchored implants have been successful in the upper jaw to provide posterior support for prostheses. This technique, introduced and developed by Brånemark to avoid bone grafting, involves placement of two to four conventional dental implants in the anterior maxilla in addition to the bilateral zygomatic implants [5–8]. Although these anterior implants are important for cross-arch stability, most studies involving the zygomatic implants focus solely on reporting the survival rate of zygomatic implants and leave out information about the regular anterior implants.

The reported overall 5-year survival rate of conventional dental implants in the edentulous maxilla is 96.7% [9]. A systematic review covering longer follow-up periods indicated a survival rate of 96.5% at 10 years and 91.2% at 20 years [10].

To the best of our knowledge, only one literature review has reported on the survival rate of conventional anterior implants placed in combination with zygomatic implants. These findings, based on 32 included studies, showed a 95.9% survival rate from among 3297 regular implants. Nevertheless, follow-up periods were variable, ranging from 1 to 84 months, and the included studies lacked homogeneity in the treatment protocols used, with variation in the number of zygomatic and conventional implants [11].

To fill in these gaps, here we evaluated the survival rate of conventional anterior implants placed in combination with zygomatic implants according to the Brånemark technique and identified risk factors for failure.

## Methods

For this longitudinal cohort study, data were collected retrospectively from patient records in the Department of Oral and Maxillofacial Surgery in East-Limburg Hospital, Genk, Belgium. Patients whose records indicated that they received zygomatic and conventional implants in the edentulous maxilla, according to Brånemark’s original technique, were included. Patients were included only if implant placement occurred between January 1, 1998, and December 31, 2018. Patients with incomplete records or no possible follow-up of at least 2 years after implant placement were excluded. All included patients were referred to one maxillo-facial surgeon specialized in zygomatic implant placement. This study was approved by the Ethics Committee of the East-Limburg Hospital.

The following variables were retrieved from the patient records at the time of implant placement: age, sex, smoking habits, parafunctions, general health, systemic diseases, existing sinus pathology, periodontal status, osteoporosis, chemotherapy, irradiation, antagonistic jaw (teeth/prothesis), and type of prothesis in the maxilla (overdenture/fixed bridge). We did not include the following variables as risk factors because of insufficient data for statistical comparison: existing sinus pathology, periodontal status, osteoporosis, chemotherapy, and irradiation. In addition, two implant-related variables, implant length and locus, were extracted from patient files for analysis.

### Radiologic examination

All patients underwent preoperative clinical and radiographic evaluation. Cone-beam computed tomography or dental computed tomography was conducted to determine the residual bone volume and desired implant positions and length. A three-dimensional evaluation of the maxilla and sinus cavity was performed.

### Surgery

Surgeries were performed with the patient under general anesthesia with nasal intubation supplemented with local anesthetic agents with vasoconstrictor (articaine, 1:100,000) for hemostasis. All procedures were carried out by the same surgeon (LV). Surgeon experience was divided into three categories for analysis as a risk factor for failure: < 5 years, 5–10 years, and ≥ 10 years.

**Fig. 1:**
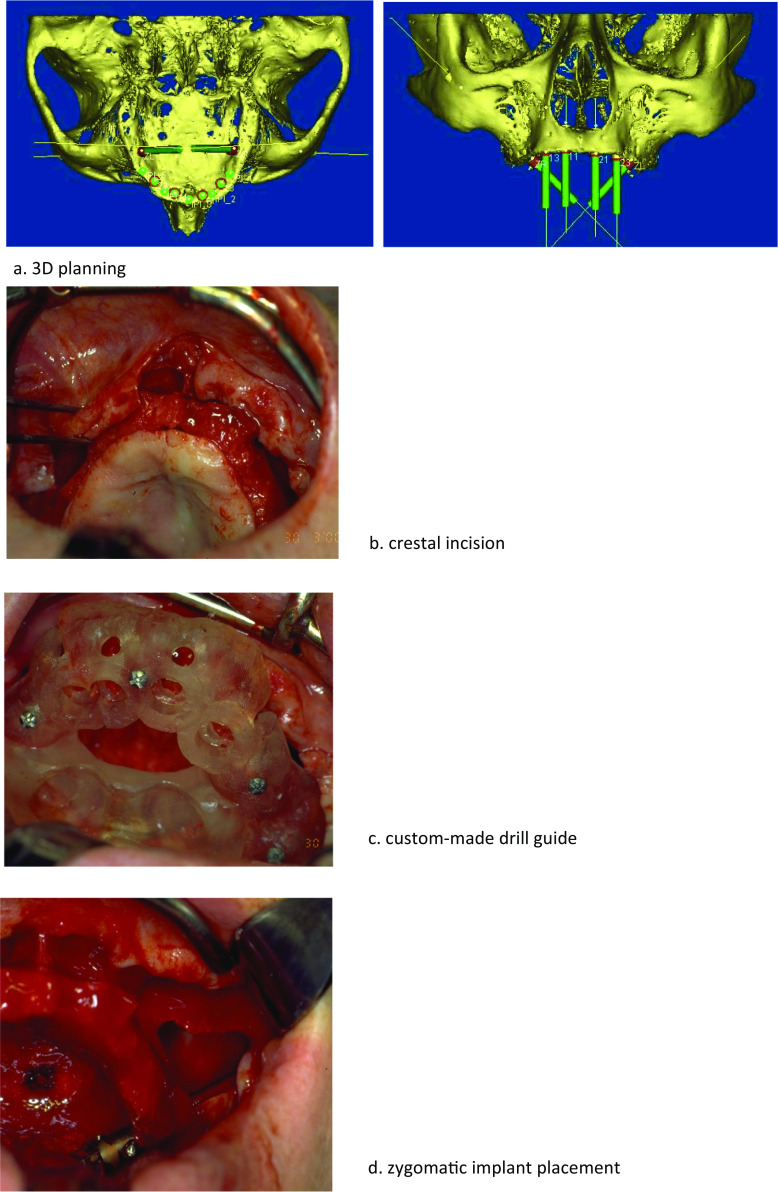

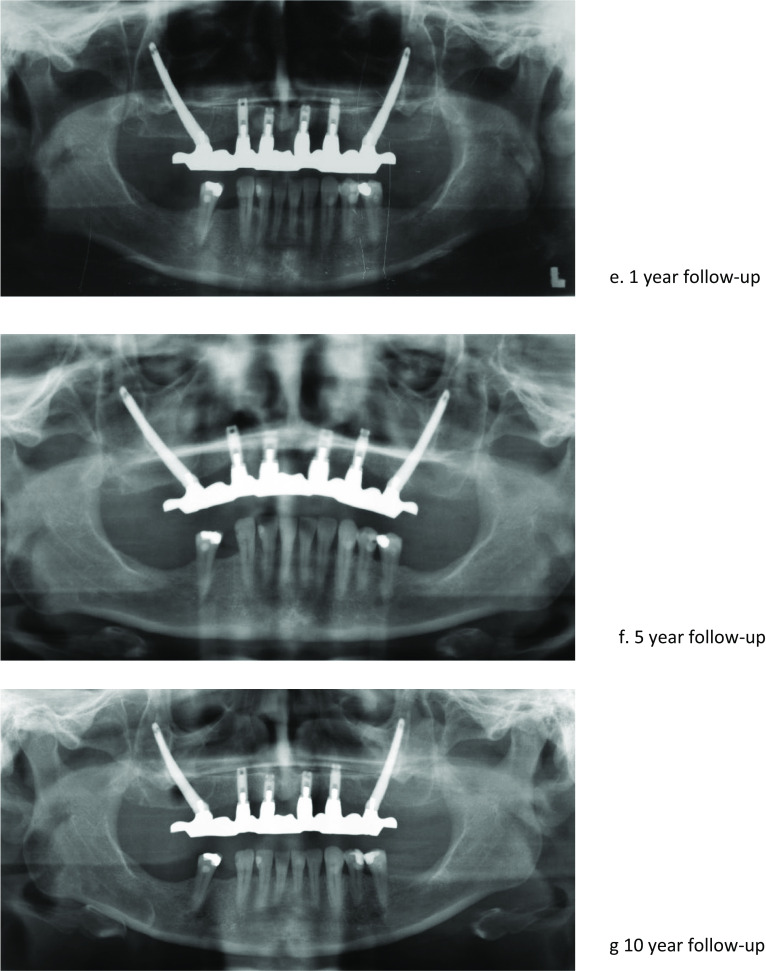
**a** 3D planning. **b** Crestal incision. **c** Custom-made drill guide. **d** Zygomatic implant placement. **e** 1-year follow-up. **f** 5-year follow-up. **g** 10-year follow-up

A mucoperiosteal crestal incision was made, and a flap was raised from the maxillary crest to the zygomatic buttress. The infraorbital nerve was identified to prevent paresthesia and other neural injuries. The palatal side of the alveolar crest also was exposed. For most patients, a custom-made drill guide was available and thus be fixated onto the bone [12]. Osteotomies for the placement of the zygomatic and conventional implants were performed according to the instructions of the manufacturer, depending on the used type of implant. After removal of surgical drill guide, zygomatic implants were placed according to either the intra-sinus technique, sinus slot technique, or the extra-sinus technique (Fig. 1). The chosen technique depended on the anatomic findings (Table 1) [13]. In cases with minor resorption of the maxilla and a small concavity of the alveolar ridge, the intra-sinus technique was preferred [14]. When the concavity of the alveolar ridge was large, the sinus slot technique was used [15]. In patients with a resorbed alveolar ridge and a large buccal concavity, the extra-sinus technique was chosen [16].

**Table 1:** Zygomatic implant techniques

Buccal concavity	Alveolar ridge	Surgical technique
Small	Present	Intra-sinus
Large	Present	Sinus slot
Large	Resorbed	Extra-sinus

In cases with only mental guiding a supracrestal incision was made in the anterior maxilla and a mucoperiosteal flap was reflected allowing visualization of the alveolar process for correct angulation. After implant placement, a cover screw was placed, and primary wound closure was obtained in the two-stage procedure, or an abutment was placed in case of one-stage procedure.

Brånemark System Zygoma implants were used with a length ranging from 30 to 52.5 mm. Analgesics (ibuprofen 600 mg three times daily) were prescribed if needed for pain. Prophylactic antibiotics were not routinely prescribed. Patients were usually discharged from the hospital the day after the procedure.

### Prosthetic procedure

For the two-stage procedure, the abutments were placed at the second-stage surgery under local anesthesia. The existing removable protheses were adapted by a dentist until final prosthetic rehabilitation was provided. For those undergoing the one-stage procedure, an acrylic provisional prothesis was provided the day of the surgery or within days after implant placement.

### Follow-up

Patients were recalled 3 and 6 months after implant placement. All treated patients were part of a follow-up program with recall every 2 years. During follow-up, clinical investigations were performed to assess infection, pain, control of plaque and inflammation, and implant stability. Radiographic evaluation of the implants was performed, and if needed, treatment was administered to resolve complications.

### Determination of implant survival

Implant survival was defined as the implant remaining in situ throughout the observation period, with or without interventions.

### Statistical analysis

The statistical package SPSS 21.0 (SPSS Inc. Chicago, IL, USA) was used for descriptive statistics and analysis. The cumulative survival rate of the conventional implants was determined with Kaplan–Meier analysis. A survival regression model was applied, with the patient as the random factor and using the Weibull distribution, which we chose for its higher likelihood than the Gaussian, exponential, lognormal, and log-logistic distributions. Differences between groups were separately corrected for each variable for simultaneous hypothesis testing according to Tukey. A *p* value of 0.05 was considered statistically significant. Statistical analysis was supervised by a senior statistician.

## Results

Records were analyzed for 72 consecutive referred patients who received this treatment from 1998 to 2018. A total of 380 implants (236 conventional anterior implants and 144 zygomatic implants) were placed. The anamnestic, clinical, and radiographic variables from each visit were obtained from patient records, and Table 2 summarizes the patient characteristics.

**Table 2:** Patient demographics

Characteristics	*n* (%)
Total patients	72
Males	37 (51.4)
Females	35 (48.6)
Age (in years)	
Mean	57.8 ± 9.2
Median	59
Range	31–78
Smoking	13 (18.1)
Non-smoking	59 (81.9)
Bruxism	6 (8.3)
No bruxism	66 (91.7)
Diabetic	2 (2.8)
Non-diabetic	70 (97.2)

The mean patient age at baseline was 57.8 years (range 31–78), and 49% were female. Sixty percent of the patients still had natural dentition in the mandible, and the rest had either fixed or removable protheses. Thirteen patients (18%) were smokers. Six patients had bruxism, and two had diabetes. One patient had received radiation in the maxillary region, and another patient had received chemotherapy because of rectal cancer. Sinus pathology was present in four patients before any implant treatment. Because of a history of cocaine abuse, an oroantral fistula was present in one patient, which was closed and left to heal prior to implant placement.

The length of conventional implants placed in the anterior maxilla ranged from 7 to 20 mm, and the most frequently placed conventional implants were 13 mm. The positions, lengths and types of anterior implants are shown in Table 3. Most anterior implants were placed in locus of the maxillary canine.

**Table 3:** Anterior implant variables

Variable	Category	*n* (%)
Implant length	7 mm	1 (0.4)
	10 mm	47 (19.9)
	11.5 mm	4 (1.7)
	13 mm	139 (58.9)
	15 mm	25 (10.6)
	16 mm	2 (0.8)
	18 mm	1 (0.4)
	20 mm	17 (7.2)
Implant locus	1 (central incisor locus)	79 (33.5)
	2 (lateral incisor locus)	38 (16.1)
	3 (canine locus)	100 (42.4)
	4 (first premolar locus)	19 (8.1)
Implant type	Brånemark System Mk III TiUnite	165 (69.9)
	Brånemark RP TiUnite	34 (14.4)
	NobelSpeedy Groovy	24 (10.2)
	Osseotite implant	4 (1.7)
	Tekka implant	4 (1.7)
	Nobel Replace Select Tapered	4 (1.7)
	Nobel parallel CC	1 (0.0)

n = number of conventional implants

In 62 patients, a custom-made drill guide (SurgiGuide®, Materialise, Leuven, Belgium) was made to transfer the preoperative plan to the patient [16]. In 10 patients, software planning was used only as a reference to determine correct implant position. Immediate loading was used in 5 patients and delayed loading after 6 months in 67 patients (Table 4). The most frequently reported construction was two zygomatic implants in combination with four anterior implants in 41 patients (Z2A4). In 10 patients, three anterior implants were placed (Z2A3), and only two anterior implants were placed in 21 patients (Z2A2).

**Table 4:** Treatment characteristics

Characteristics	*n* (%)
Surgeon experience (in years)	
< 5 years	22 (30.6)
5–10 years	19 (26.4)
≥ 10 years	31 (43.0)
Loading	
Immediate	5 (6.9%)
Delayed	67 (93.1)
Number of anterior implants	
2	21 (29.2)
3	10 (13.9)
4	41 (56.9)
Type of prosthesis	
Fixed prosthesis	44 (61.1)
Overdenture	28 (38.9)

The mean follow-up time was 12.9 years (range 2.2–21.9 years), with a standard deviation of 5.5 years. We censored 102 implants because 12 patients died during follow-up (43 implants) and another 17 patients (59 implants) were lost to follow-up for other reasons (e.g., follow-up at another clinic, lost to contact, medical reasons).

**Fig. 2:**
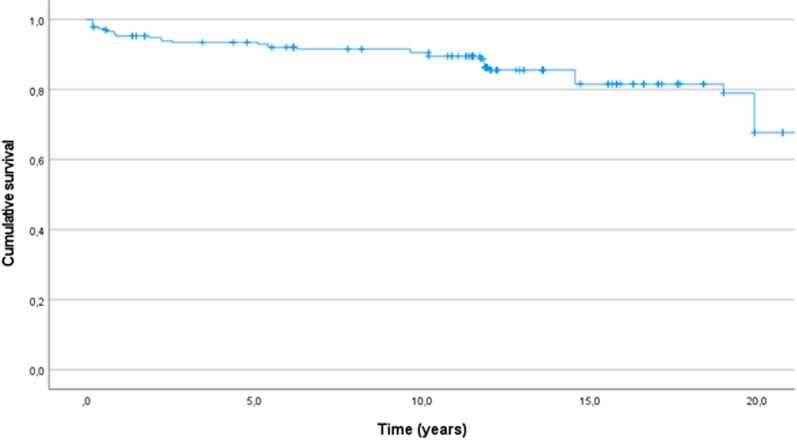
Kaplan–Meier curve for overall anterior implant survival over the course of 20 years

The Kaplan–Meier curve (Fig. 2) shows an overall cumulative anterior implant survival rate of 95.3% at 1 year, 94.8% at 2 years, 93.0% at 5 years, 90.5% at 10 years, 81.6% at 15 years, and 67.7% at 20 years.

For every variable, the relative and absolute frequency of patients and implants is presented in Table 5. Survival over the course of 20 years for every category is shown.

**Table 5:** Patient and treatment characteristics and survival

Variable	Category	Number of patients	Relative frequency of patients (%)	Number of anterior implants	Relative frequency of anterior implants (%)	1-year survival (%)	2-year survival (%)	5-year survival (%)	10-year survival (%)	15-year survival (%)	20-year survival (%)
Age, baseline	≤ 60	44	61.1	144	61.0	94.4	93.6	93.6	89.1	74.0	59.9
	> 60	28	38.9	92	39.0	96.7	96.7	93.2	93.6	93.6	84.8
Sex	Female	35	48.6	117	49.6	96.5	96.5	93.5	90.3	88.9	74.7
	Male	37	51.4	119	50.4	94.1	93.2	93.2	90.6	73.9	63.3
Smoking	No	59	81.9	196	83.1	98.0	98.0	94.9	91.7	88.7	64.6
	Yes	13	18.1	40	16.9	87.5	87.5	87.5	87.5	87.5	NA
Bruxism	No	66	91.7	217	91.9	97.2	96.7	95.2	92.0	82.0	70.4
	Yes	6	8.3	19	8.1	73.7	73.7	73.7	73.7	73.7	NA
Indication	First treatment	61	84.7	203	86.4	95.6	95.0	95.0	93.9	84.3	70.0
	Retreatment	11	15.3	33	14.0	93.5	93.5	83.9	68.6	NA	NA
Mandible	Teeth	43	59.7	143	60.6	93.6	92.8	90.4	85.3	76.9	58.8
	Prosthesis	29	40.3	93	39.4	97.8	97.8	97.8	97.8	87.6	81.7
Surgeon experience	< 5 years	22	30.6	84	35.6	92.9	92.9	92.9	91.6	78.6	65.3
	5–10 years	19	26.4	76	32.2	100.0	100.0	95.5	92.4	89.3	89.3
	≥ 10 years	31	43.0	76	32.2	93.4	92.1	92.1	86.9	83.7	NA
Number of anterior implants	2 (Z2A2)	21	29.2	42	17.8	100.0	97.5	97.5	94.9	NA	NA
	3 (Z2A3)	10	13.9	30	12.7	90.0	90.0	90.0	90.0	84.7	84.7
	4 (Z2A4)	41	56.9	164	69.5	95.0	95.0	93.0	89.6	79.8	64.7
Type of prosthesis	Overdenture	44	61.1	133	56.4	93.2	92.4	90.0	86.5	82.1	63.8
	Fixed bridge	28	38.9	103	43.6	98.0	98.0	98.0	95.7	82.2	82.2

*NA* not applicable

Table 6 shows survival based on implant length and locus. Absolute and relative frequency of implants per variable is depicted.

**Table 6:** Implant characteristics and survival

Variable	Category	Number of anterior implants	Relative frequency of anterior implants (%)	Survival (%)					
				1 y	2 y	5 y	10 y	15 y	20 y
Implant length	≤ 10 mm	48	20.3	87.2	87.2	87.2	78.0	56.5	28.3
	> 10 mm	188	79.7	97.3	96.7	95	93.8	89.6	82.2
Implant locus	1 (central incisor)	79	33.5	93.6	93.6	92.2	90.8	76.3	70.0
	2 (lateral incisor)	38	16.1	92.1	92.1	92.1	88.7	88.7	44.3
	3 (canine)	100	42.4	97.0	95.9	93.8	89.4	82.0	63.5
	4 (first premolar)	19	8.1	100	100	100	100	100	100

The survival regression model using Weibull distribution revealed a significant survival regression coefficient of 1.309 (*p* = 0.015) for bruxism and of 0.906 (*p* = 0.030) for type of prosthesis used in the maxilla, in favor of fixed prostheses compared with overdentures. Additionally, a significant survival regression coefficient 0.563 (*p* = 0.048) was found for implant length in favor of anterior implants > 10 mm (Table 7).

**Table 7:** Survival regression coefficient per variable

Variable	Comparison	Survival regression coefficient	*p*
Age at baseline	> 60 vs. ≤ 60 years	0.394	0.319
Sex	Female vs. male	0.370	0.317
Smoking	No vs. yes	0.221	0.639
Bruxism	No vs. yes	1.309	0.015*
Indication	First- vs. re-treatment	0.570	0.226
Mandible	Prothesis vs. teeth	0.680	0.078
Surgeon experience	5–10 years vs. < 5 years	0.156	0.784
	5–10 years vs. ≥ 10 years	0.434	0.631
	≥ 10 years vs. < 5 years	− 0.278	0.784
Anterior implants	2 vs. 3 anterior implants	0.662	0.630
	2 vs. 4 anterior implants	0.551	0.618
	3 vs. 4 anterior implants	0.112	0.977
Maxilla	Fixed bridge vs. overdenture	0.906	0.030*
Implant length	> 10 mm vs. ≤ 10 mm	0.563	0.048*
Implant locus	1 vs. 2	− 0.012	0.999
	1 vs. 3	− 0.102	0.915
	1 vs. 4	− 0.627	0.738
	2 vs. 3	− 0.090	0.991
	2 vs. 4	− 0.615	0.779
	3 vs. 4	− 0.525	0.831

*Significant

## Discussion

In recent decades, many studies have focused on the success and long-term survival of zygomatic implants as a predictable solution for rehabilitation of the atrophic maxilla [5, 17–26]. Of the 66 studies included in a recent systematic review of survival and complications with zygomatic implants, only 13 reported the failure period of conventional implants placed in the premaxilla [27].

To advise patients accurately, practitioners must have reliable information regarding the long-term survival rates of different treatment options. In this study, the cumulative survival rates of anterior implants placed in combination with the zygomatic implants were 90.5% at 10 years, 81.6% at 15 years, and 67.7% at 20 years. As noted, data comparing long-term survival rates are scarce, and no studies have reported follow-up longer than 7 years for anterior implants placed in combination with zygomatic implants [27].

For the general survival rate of conventional implants, only 4 of 21 included studies in a systematic review reported a survival rate up to 20 years [10]. The survival rates we identified here are lower than those reported by Moraschini et al. for conventional dental implants in general. However, our findings are acceptable taking into consideration the challenges of the resorbed maxilla. Furthermore, it is important to note that no exclusion criteria based on patient characteristics were applied. Patients with risk factors such as smoking, diabetes, systemic disease, oncologic history or sinus pathology were included. Therefore, realistic survival numbers could be presented, because nowadays, also patients who present with one or more risk factors are eligible for prosthetic oral rehabilitation.

Another factor that needs to be taken into consideration is the age of the patients. Most patients received treatment around the age of 60 years; therefore, at follow-up times of 15–20 years they might be more susceptible to implant loss caused by peri-implantitis due to old age and therefore reduced ability to maintain proper oral hygiene.

Mean follow-up time was 12.1 years, therefore there were less patients in follow-up in the last 10 years. This might explain the bigger drops in survival we observed towards the end of the observational period compared to the more gradual decline at the start.

When we analyzed each risk factor separately, we found no significant survival regression coefficient for failure of anterior implants at older age, in agreement with a recent systematic review [28]. Chrcanovic et al. reported that most studies found no significant effect of patient sex on implant failure, also in accord with our current findings [29]. Smoking is a known risk factor for early implant failure, and we found here that all implants that failed in smokers did so in the first months after placement [30]. However, in our study no significant difference in survival could be found for smoking.

Bruxism is a common parafunctional habit that is generally accepted to determine implant and prosthetic overload and is a plausible contributor to dental implant technical and biological failure [31, 32]. Comparisons of implants placed in the anterior maxilla in those with and without bruxism have shown a statistically significant difference in survival [33]. Our study confirmed this difference, with a strong survival regression coefficient of 1.309 for bruxism as factor in implant failure. The Kaplan–Meier analysis showed that all related failures occurred within the first months after implant placement. The implication is that for patients with waking or nocturnal bruxism, measures are needed to increase the chance of implant survival. For nocturnal bruxism, a night guard is recommended. Moreover, all implant failures occurred in bruxers who received 3 or 4 anterior implants and no failure in bruxers with 2 anterior implants. Finite element analysis in literature has shown that the placement of just 2 anterior implants is more favorable compared to 4, considering stress applied on the alveolar bone under lateral loading [34]. This finding suggests, although counterintuitive, that the placement of 2 anterior implants is a better option over 3 or 4 anterior implants, especially in patients with bruxism.

Bruxism was only scored at the time of implant placement and no additional analysis was made for patients who might develop bruxism after prothesis placement.

In patients receiving implants in the maxilla who have a history of previously failed implant rehabilitation, a significantly higher failure rate could be expected [35, 36]. However, we found no significantly lower survival of anterior implants in these patients. Therefore, re-implantation can remain a preferred treatment that offers superior occlusal stability, nutritional intake, masticatory efficiency, and oral health-related quality of life [37–40]. Nevertheless, the recommendation remains that patients should be informed of all alternative treatment modalities before considering a second or third implant-supported prosthesis [41].

The presence of antagonistic teeth in the lower jaw was not a significant risk factor for failure in the current study. Multiple reports have described the presence of natural occluding teeth as a risk factor for antagonistic prosthesis-bearing implant success, but this feature cannot be considered to be a contraindication for rehabilitation of the atrophic maxilla [42–44].

Only one maxillo-facial surgeon, specialized in zygomatic implant placement in this center, performed all surgeries in included patients, and we analyzed outcomes based on 5-year increments of this surgeon’s years of experience. A variety of complications are associated with zygomatic implant placement in the literature, and the procedure preferably should be executed by an experienced clinician [27, 45]. One meta-analysis showed that surgical experience based on the number of implants placed significantly affected implant failure [46]. We did not, however, find a significant effect of years of experience for this surgeon on anterior implant survival.

The number of conventional implants used in combination with the two zygomatic implants also did not affect implant survival. Based on this result, we conclude that if sufficient bone volume is available only for placement of two anterior implants, this treatment option is a viable one.

Five of our patients underwent immediate loading, and none of the 14 implants placed in these patients failed. This sample size was insufficient, however, for a comparison with implant survival in patients who received delayed loading. Nonetheless, multiple studies have demonstrated immediate loading to be a reliable option [47, 48].

Previous reports indicate that implant loss rates for fixed restorations are significantly lower than those with a removable prosthesis [9]. Our findings agree with these earlier results, showing a significant survival regression coefficient of 0.906 (*p* = 0.03) in favor of implants bearing a fixed prosthesis. Failure for the removable prothesis cannot be explained by the lack of cross-arch stability, because the overdentures in almost all patients were based on a metal bar connecting all the implants. However, overdentures might have been preferably used more often in patients with a higher grade of atrophy, which made the shorter implants placed in these cases more prone to failure.

Implants with length ≤ 10 mm were associated with significantly lower implant survival, with a survival regression coefficient of 0.563 (*p* = 0.048). Previous findings have been inconclusive regarding failure rates with short vs. long implants in the maxilla, although most studies have focused solely on short implant survival in the posterior maxilla [49–52]. We suggest that if there is insufficient anterior bone to place implants > 10 mm, treatment with quad zygoma might be a viable option. The implant locus in the current study did not significantly affect survival rate or risk. Survival is expected to be lower in the posterior maxilla compared with the anterior maxilla, but no gradation has been found in the results of this study [53].

The limitations of this study include the involvement of only one center and the retrospective design. The generalizability of this study might be compromised by the limited sample size, the poor control of patient inclusion criteria due to the retrospective design, and the use of mainly one type of conventional implant. As mentioned, variables that can change over the course of time were only retrieved from the patient records at the time of implant placement. Therefore, the effect of changes in smoking habit or bruxism was not analyzed. Although we sought to minimize loss to follow-up for the recall every 2 years, a few patients were unable or unwilling to attend. The dropout rate (17 of 72 patients, 23%) must be considered as acceptable over this long follow-up period, but has to be taken into account when analyzing the result of this study.

We plan to analyze zygomatic implant success and risk factors for failure in future work.

## Conclusions

The main objective of this study was to report the survival rate of conventional anterior implants placed in combination with zygomatic implants according to the Brånemark technique, and to identify risk factors for failure. Kaplan–Meier analysis showed an acceptable overall cumulative survival rate of 95.3% at 1 year, 94.8% at 2 years, 93.0% at 5 years, 90.5% at 10 years, 81.6% 15 years, and 67.7% at 20 years. Significant risk factors for failure were bruxism and implants bearing overdentures as opposed to fixed dentures. Implants shorter than 10 mm had a higher chance of failure compared with longer options. Following the results of this study, whenever there is only sufficient bone volume in the anterior maxilla to place 2 anterior implants in combination with 2 zygomatic implants, this can be a valid treatment option. Further studies including randomized clinical trials of the influence of treatment variables on survival are suggested to confirm the presented outcomes.

## Data Availability

The datasets used and/or analyzed during the current study are available from the corresponding author on reasonable request.

## Abbreviations

Z2A4: Two zygomatic implants in combination with four anterior conventional implants
Z2A3: Two zygomatic implants in combination with three anterior conventional implants
Z2A2: Two zygomatic implants in combination with two anterior conventional implants
